# Epidemiology and survival outcomes of patients with primary intraocular lymphoma: a population-based analysis

**DOI:** 10.1186/s12886-022-02702-6

**Published:** 2022-12-13

**Authors:** Lin-feng He, Jin-di Zhang, Xin-xin Chen, Rui-li Wei

**Affiliations:** Department of Ophthalmology, Changzheng Hospital of Naval Medicine University, 415 Fengyang Road, Shanghai, China

**Keywords:** Primary intraocular lymphoma, SEER program, Epidemiology, Prognosis

## Abstract

**Background:**

Primary intraocular lymphoma (PIOL) is a rare malignancy with a poor prognosis, but its optimal therapy remains unclear. Herein, we aimed to analyze the epidemiology and survival outcomes of PIOL patients based on a population-based cancer registry in the United States.

**Methods:**

Patients diagnosed with PIOL between 1992 and 2018 were identified from the Surveillance Epidemiology and End Results program. The patients were divided into two groups: those aged < 60 years and ≥ 60 years. We used the chi-squared test to analyze the differences between the two groups. Descriptive analyses were performed to analyze epidemiological characteristics and treatment. The likely prognostic factors were analyzed by Kaplan–Meier curves and Cox proportional hazards models.

**Results:**

The overall incidence of PIOL was 0.23/1,000,000, which was steadily increasing from 1992 to 2018, with an annual percentage change of 2.35. In total, 326 patients (mean age, 66.1 years) with PIOL were included in this study, 72.1% were aged ≥ 60 years, 84.4% were White, and 60.4% were female. The most common pathological type was diffuse large B-cell lymphoma (DLBCL), but in patients aged < 60 years, extranodal marginal zone lymphoma of mucosa-associated lymphoid tissue was the most common. The disease-specific survival rates were 74.2% and 61.5% 5 and 10 years after diagnosis, respectively. Survival analysis found that surgery, radiation, and chemotherapy did not lead to better prognosis.

**Conclusions:**

PIOL is a rare disease with poor prognosis, and its incidence has been increasing for nearly 30 years. It usually affects people aged ≥ 60 years, and DLBCL is the most common pathological type of PIOL. Patients aged < 60 years and with non-DLBCL type have improved survival. Survival of PIOL has improved in recent years.

## Background

Primary intraocular lymphoma (PIOL) is a rare heterogeneous malignancy and considered a subset of primary central nervous system lymphoma (PCNSL), with lymphoma cells initially existing only in the eyes [1, 2]. As PIOL is a rare subset of PCNSL, most of its epidemiology data are deduced from studies on PCNSL, whose incidence rate has increased fivefold over the past 40 years, with its peak incidence occurring in those aged 75–84 years [3–5]. Between 15 to 25% of PCNSL patients have or will eventually develop ocular lymphoma [5]. Approximately 60–80% of PIOL patients develop central nervous system (CNS) disease within a mean of 29 months, which causes extremely poor prognosis [6–8]. PIOL primarily arises from the retina and vitreous body, in a few cases arising from the uveal and optic nerve [9, 10]. The most common histological subtype of PIOL is diffuse large B-cell lymphoma (DLBCL); rarely, T-cell lymphoma can be detected [11]. Patients often complain of floaters and blurred vision, and less commonly of red eye, photophobia, and ocular pain [12–14]. Establishing the diagnosis of PIOL is challenging as it usually presents as masquerade syndrome, imitates chronic uveitis, and may even respond to steroid treatment [15, 16]. The mean time from first symptom onset to definitive diagnosis ranges from 6 to 40 months [17, 18]. Histopathological analysis remains a cornerstone of diagnosing PIOL; however, rapid cell degeneration, small number of cells, and interfering impurity in the samples continue to make the analysis difficult [19]. The accuracy of PIOL diagnosis has been improved via immunocytochemistry, biochemical finding of elevated interleukin (IL)-10 levels with an IL-10:IL-6 ratio > 1.0, flow cytometry, and cellular microdissection with polymerase chain reaction amplification [16, 20]. When a diagnosis of PIOL is established, a patient should be referred to an oncologist, and a complete system review, especially CNS evaluation, should be performed [21].

Due to its rarity, the understanding of PIOL is mainly derived from small-sample retrospective studies [5–7]. Few epidemiological studies have reported on the incidence, demography, clinicopathology, and survival outcomes of PIOL. The Surveillance, Epidemiology, and End Results (SEER) database, which collects the incidence and survival information of cancer patients covering almost 28% of the population in the United States (US), provides valuable information on tumor characteristics and survival outcomes and is an especially vital resource for studies of rare cancers [22, 23].

## Methods

### Data source and study population

Study data were obtained from the SEER registry of the National Cancer Institute using the SEER*Stat software (version 8.4.0.1). To increase the representativeness of this study, PIOL patients were extracted from two databases from SEER: those diagnosed between 2000 and 2018 were extracted through the SEER 18 registry data [24], and patients diagnosed between 1992 and 1999 were extracted through the SEER 13 registry data [25]. The International Classification of Diseases for Oncology histological codes (9590–9599, 9650–9669, 9670–9729, 9735, 9737, 9738, 9811–9815, 9823, 9827, and 9837) were used for lymphoma combined with primary site codes (C69.2, C69.3, C69.4 and C69.9) to identify lymphoma primarily limited intraocular. The site eye, not otherwise specified (NOS) (C69.9), was used to refer to the vitreous. The patients were diagnosed by microscopic confirmation. The exclusion criteria were as follows: (1) patients who had prior cancer diagnoses, (2) patients diagnosed at autopsy or death certificate or without active follow-up, (3) patients who survived for 0 months or whose survival time was unknown, and (4) patients aged < 16 years in survival analysis, because the treatment modalities for children differ from those for adults (only two patients). As the SEER database is publicly available and all information is anonymized, this study was exempt from any institutional review board approval.

### Study variables

The following variables were extracted from the SEER: patient ID, age at diagnosis, year of diagnosis, sex (female and male), race (White and others), primary site, laterality, histological type, surgery at the primary site (yes, no/unknown), radiation code (yes, no/unknown), chemotherapy code (yes, no/unknown), cause of death, survival month, Ann Arbor stage (AAS), and vital status.

The annual incidence of PIOL from 1992 to 2018 was calculated from the SEER 13 registry data to study the tendency of the incidence, and all incidence rates were standardized to the 2000 US standard population.

### Statistical analyses

The incidence rates were calculated per 1,000,000 persons and were age-adjusted to the standard population of the US in 2000 using SEER*Stat version 8.4.0.1. The annual percentage change (APC) and 95% confidence interval (CI) were also calculated using the SEER*Stat software. The incidence of PIOL was statistically compared based on age, sex, and race using the chi-squared test.

Descriptive statistics were computed for all variables. All variables were converted to categorical variables, and presented as frequencies. We divided the patients into two groups, aged < 60 years and ≥ 60 years, and evaluated the differences in patients’ demographic and clinicopathological characteristics using the chi-squared test. Statistical significance was set to *P* < 0.05. Kaplan–Meier curves were used to analyze disease-specific survival (DSS), and the differences were estimated using the log-rank test. Univariate Cox proportional hazards models were applied in survival analysis. *P* values < 0.05 were considered statistically significant. Statistical analyses were performed using the Statistical Package for the Social Sciences (SPSS version 26.0, IBM SPSS statistics, IBM Corporation, Armonk, NY, USA) and R software (version 4.2.1).

## Results

### Incidence of PIOL

**Fig. 1:**
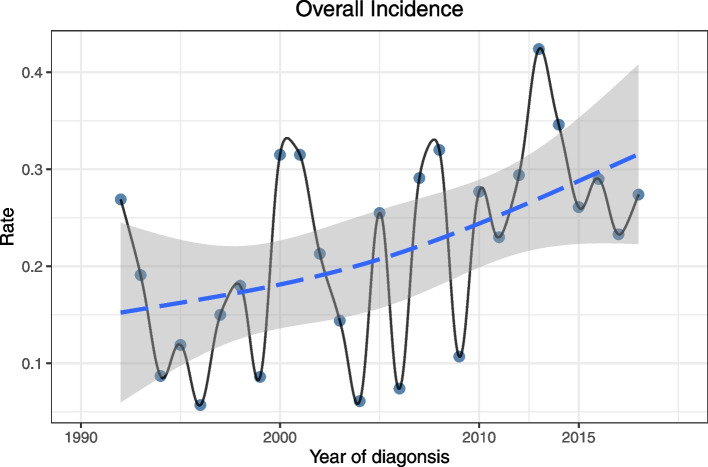
Incidence of PIOL from 1992 to 2018 adjusted to the 2000 standard United States population. PIOL, primary intraocular lymphoma

The overall incidence of PIOL was 0.23, which steadily increased from 1992 to 2018, with an APC of 2.35 (95% CI, 0.355–4.393; *P* < 0.05) (Fig. 1). The age-adjusted incidences of PIOL were 0.27 in 1992 and 0.19 in 1993. The incidences were 0.23 and 0.27 in 2017 and 2018, respectively. The incidence of PIOL increased with age, with the incidence significantly higher in patients aged ≥ 60 years (1.07) than in patients < 60 years (0.06). The incidence in men (0.24) was slightly higher than that in women (0.21), but the difference was not significant. Among the White population, the incidence was 0.24, which was significantly higher than that among other races (0.18) (Table 1).

**Table 1:** Incidence rate from 1992 to 2018

	Incidence rate	Incidence rate ratio(95%CI)	*P*
**Overall**	0.225		
**Age**			
<60	0.058	Ref	<0.001
≥ 60	1.073	17.66(13.047–23.905)	
**Sex**			
Male	0.241	Ref	
Female	0.214	1.104(0.857–1.422)	0.444
**Race**			
White	0.237	Ref	0.004
Others^a^	0.182	0.613(0.439–0.855)	

Others^a^: Black,American Indian/AK Native,Asian/Pacific Islander,and unknown

### Clinicopathological characteristics

In total, data from 326 patients were extracted from the SEER database, 197 (60.4%) of whom were female and 275 (84.4%) were White. The mean age of the patients at diagnosis was 66.1 ± 14.2 years, with a wide range of 8–97 years, and 235 (72.1%) patients were aged ≥ 60 years. The vitreous (68.7%) was the most common primary site, followed by the ciliary body (20.2%), choroid (7.4%), and retina (3.7%). Among diverse pathological types, the most common type was DLBCL (30.4%), followed by mucosa-associated lymphoid tissue (MALT) lymphoma (27%), others/unclassified types (25.5%), and non-Hodgkin lymphoma (NHL) (17.2%). The pathological types between the groups aged < 60- and ≥ 60 were significantly different. In the younger group, MALT lymphoma (40.7%) was the most common pathological type, whereas in the elderly group, DLBCL (34.5%) was the most common. Based on AAS, patients with stage I and II were the most common, accounting for 64.1%, and 9.5% of the patients had stage III and IV. The remaining patients (26.4%) had unknown stage. More unilateral lesions (84%) than bilateral lesions (13.5%) were observed in patients at first presentation, and laterality was unknown in 2.5% of the patients. Patients’ baseline characteristics are summarized in Table 2.

**Table 2:** Demographic and clinical characteristics of patients with primary intraocular lymphoma

Variables	Total	<60	≥ 60	P
Number of patients(%)	326	91	235	
**Age**				
Mean (SD)	66.1(14.2)	48.5(10.4)	72.9(8.6)	
Median [Min, Max]	67.5[8,97]	52[8,59]	72[60–97]	
**Year of diagnosis**				0.059
1992–2002	76(23.3)	18(19.8)	58(24.7)	
2003–2012	138(42.3)	48(52.7)	90(38.3)	
2013–2018	112(34.4)	25(27.5)	87(37)	
**Sex**				0.208
Male	129(39.6)	41(45.1)	88(37.4)	
Female	197(60.4)	50(54.9)	147(62.6)	
**Race**				0.105
White	275(84.4)	72(79.1)	203(86.4)	
Others^a^	51(15.6)	19(20.9)	32(13.6)	
**Laterality**				0.202
Unilateral	274(84)	78(85.7)	196(83.4)	
Bilateral	44(13.5)	13(14.3)	31(13.2)	
Unknown	8(2.5)		8(3.4)	
**Primary site**				0.185
Retina	12(3.7)		12(5.1)	
Choroid	24(7.4)	7(7.7)	17(7.2)	
Ciliary body	66(20.2)	19(20.9)	47(20)	
Vitreous	224(68.7)	65(71.4)	159(67.7)	
**Pathological type**				0.003
DLBCL	99(30.4)	18(19.8)	81(34.5)	
MALT	88(27)	37(40.7)	51(21.7)	
NHL, NOS	56(17.2)	13(14.3)	43(18.3)	
Other/unclassified^b^	83(25.5)	23(25.3)	60(25.5)	
**Ann arbor stage**				0.839
I to II	209(64.1)	58(63.7)	151(64.3)	
III to IV	31(9.5)	10(11)	21(8.9)	
Unknown	86(26.4)	23(25.3)	63(26.8)	
**Surgery**				0.153
No	232(71.2)	70(76.9)	162(68.9)	
Performed	94(28.8)	21(23.1)	73(31.1)	
**Radiotherapy**				0.757
No/unknown	180(55.2)	49(53.8)	131(55.7)	
Performed	146(44.8)	42(46.2)	104(44.3)	
**Chemotherapy**				0.978
No/unknown	201(61.7)	56(61.5)	145(61.7)	
Performed	125(38.3)	35(38.5)	90(38.3)	

Others^a^: Black,American Indian/AK Native,Asian/Pacific Islander,and unknown

Other/unclassified^b^: Malignant lymphoma,Mantle cell lymphoma, Burkitt lymphoma,Follicular lymphoma, Peripheral T-cell lymphoma, Anaplastic large cell lymphoma, Chronic lymphocytic leukemia/small lymphocytic lymphoma

*NHL* Non–Hodgkin lymphoma, *NOS* Not otherwise specified

### Survival analysis

**Fig. 2:**
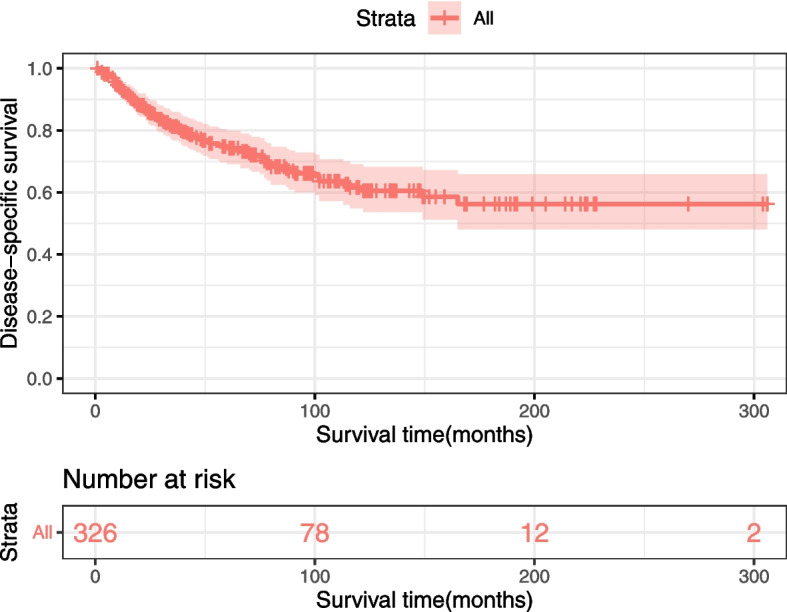
Disease-specific survival of PIOL for all patients. PIOL, primary intraocular lymphoma

**Fig. 3:**
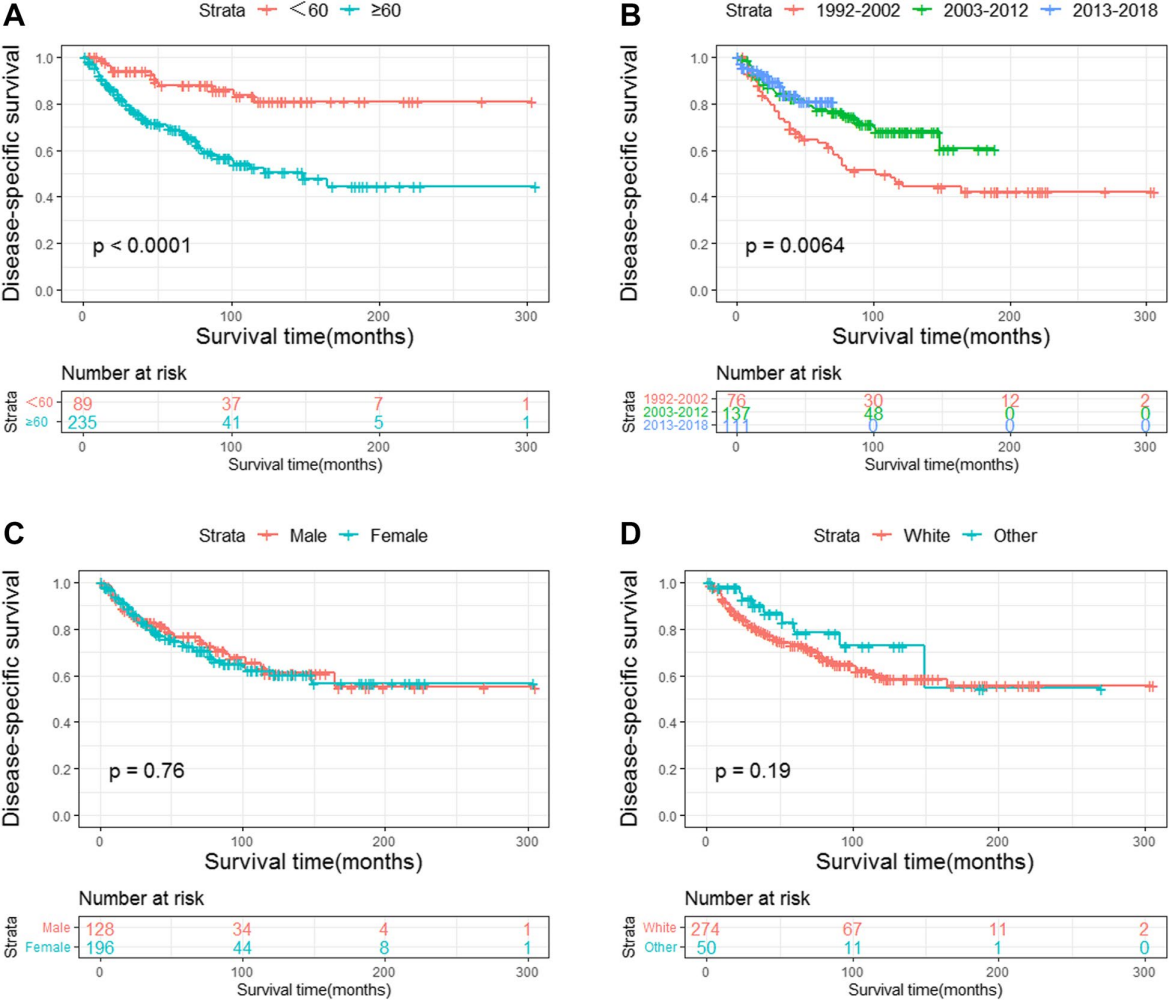
Disease-specific survival in PIOL according to age, diagnosis year, sex, and race. Disease-specific survival according to (**a**) age, (**b**) year of diagnosis, (**c**) sex, and (**d**) race

**Fig. 4:**
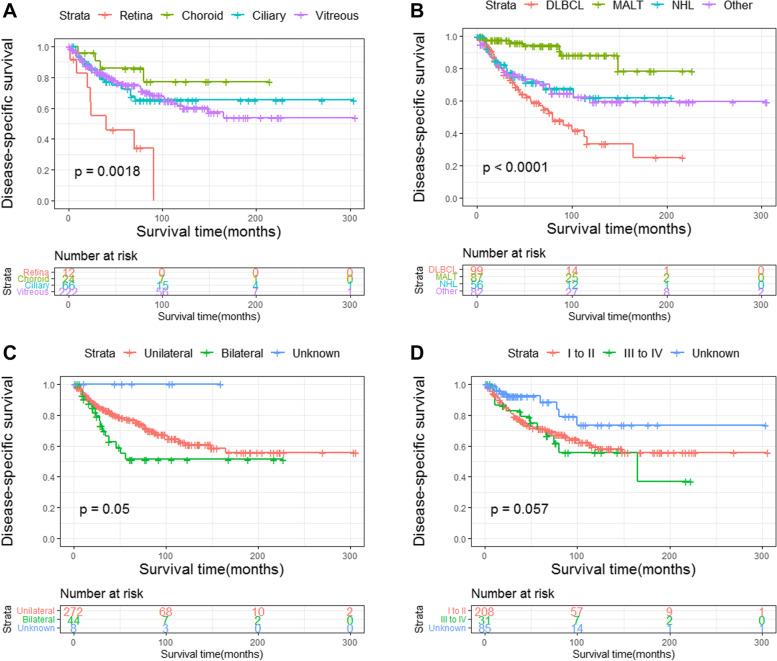
Disease-specific survival in PIOL according to primary site, histological subtype, laterality, and AAS. Disease-specific survival according to (**a**) primary site, (**b**) histological subtype, (**c**) laterality, and (**d**) AAS. AAS, Ann Arbor stage; PIOL, primary intraocular lymphoma

**Fig. 5:**
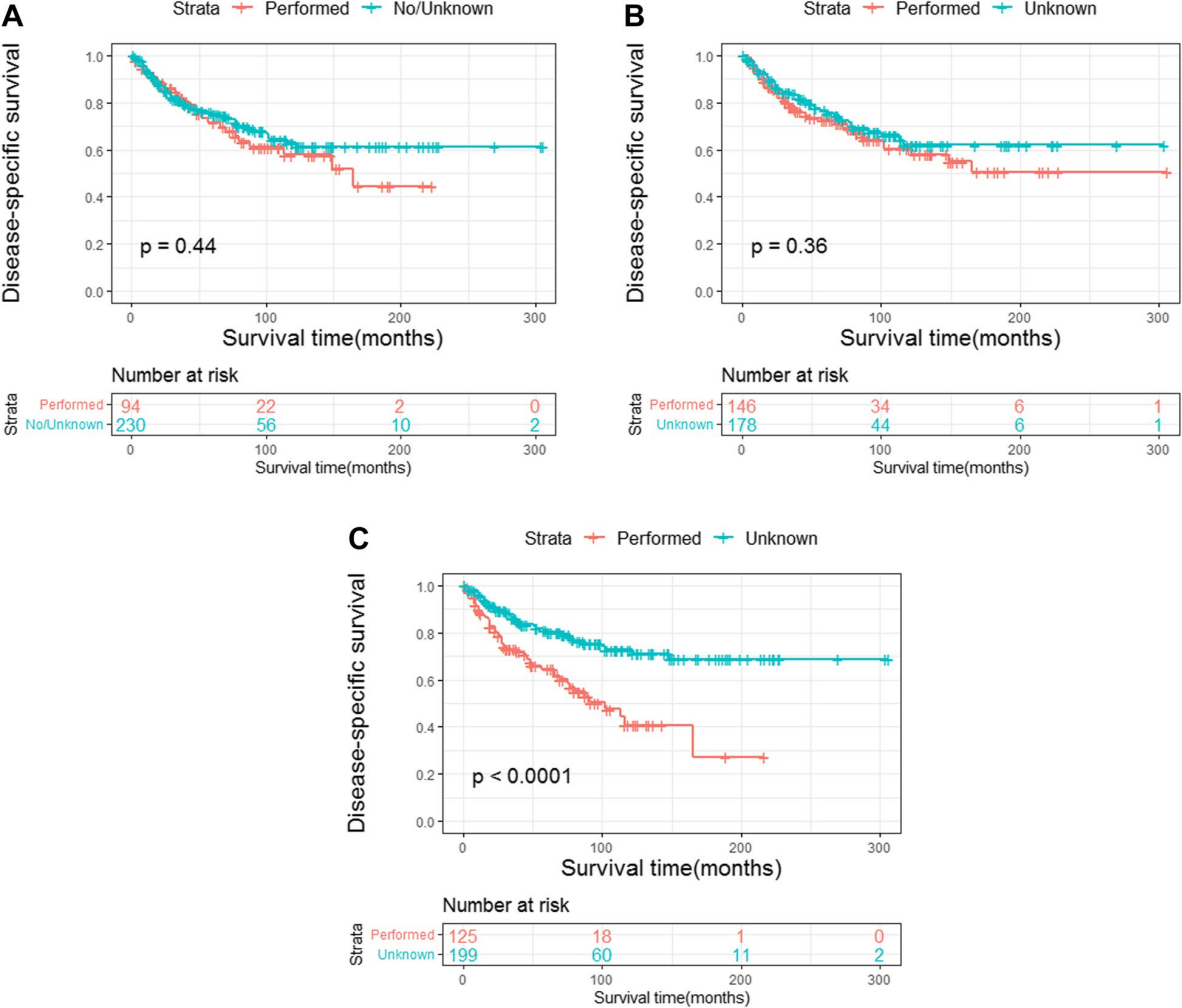
Disease-specific survival of PIOL according to surgery, radiation, and chemotherapy. Disease-specific survival according to (**a**) surgery, (**b**) radiation, (**c**) chemotherapy. PIOL, primary intraocular lymphoma

The DSS of all PIOL patients is shown in Fig. 2. Up to 156 patients died by the end of follow-up, and 90 of them died of PIOL. The 1-, 5-, and 10-year DSS rates were 92.9%, 74.2%, and 61.5%, respectively. Survival curves stratified by age, years of diagnosis, sex, race, laterality, primary site, pathological type, AAS, and treatment modality were constructed according to the Kaplan–Meier survival analysis. The analysis revealed that age ≥ 60 years was significantly associated with poor DSS (Fig. 3a). The DSS rates of patients diagnosed in 2003 − 2012 and 2013 − 2018 were significantly higher than that of patients diagnosed in 1992–2002 (both *P* < 0.05) (Fig. 3b). However, sex (Fig. 3c) and race (Fig. 3d) had no effect on DSS. According to primary sites, patients with lymphoma in the retina had the worst prognosis, whereas patients with lymphoma in the choroid had longer DSS (Fig. 4a). Among the various histological subtypes of PIOL, MALT lymphoma was associated with better DSS than other subtypes, and DLBCL had the worst DSS (Fig. 4b). The survival analysis revealed that laterality and AAS had no effect on DSS (Fig. 4c and d). In terms of treatment strategies, surgery (Fig. 5a), radiation (Fig. 5b), and chemotherapy (Fig. 5c) did not lead to better prognosis.

The whole cohort was analyzed using log-rank tests and univariate Cox proportional hazards models, which revealed that age, years of diagnosis, laterality, primary site, pathological type, and chemotherapy had an effect on DSS. The result of the log-rank tests stratified by age is shown in Table 3. Variables that exhibited *P* < 0.05 in the univariate Cox regression analysis were included in the multivariable Cox regression analysis, identified that age, pathological type, and chemotherapy were independent prognostic factors for the DSS of PIOL (Table 4).

**Table 3:** The results of the log-rank test

Variables	Total	<60	≥ 60
**Age**	< 0.001		
<60			
≥ 60			
**Year of diagnosis**	0.006	0.329	0.035
1992–2002			
2003–2012			
2013–2018			
**Sex**	0.757	0.4	0.794
Male			
Female			
**Race**	0.188	0.091	0.785
White			
Others			
**Laterality**	0.05	0.03	0.072
Unilateral			
Bilateral			
Unknown			
**Primary site**	0.002	0.946	0.018
Retina			
Choroid			
Ciliary body			
Vitreous			
**Pathological type**	< 0.001	0.347	< 0.001
DLBCL			
MALT			
NHL, NOS			
Other/unclassified^b^			
**Ann arbor stage**	0.057	0.22	0.032
I to II			
III to IV			
Unknown			
**Surgery**	0.44	0.949	0.521
NO			
Performed			
**Radiotherapy**	0.361	0.147	0.715
No/unknown			
Performed			
**Chemotherapy**	< 0.001	0.001	0.001
No/unknown			
Performed

**Table 4:** The results of the univariate and multivariate Cox regression analysis

Variables	Univariate analysis		Multivariate analysis	
	HR (95% CI)	*P* value	HR (95% CI)	*P* value
**Age**		< 0.001		< 0.001
<60	Ref		Ref	
≥ 60	3.508(1.904–6.464)		3.146(1.699–5.826)	
**Year of diagnosis**		0.008		0.054
1992–2002	Ref			
2003–2012	0.524(0.333–0.826)			
2013–2018	0.473(0.251–0.89)			
**Sex**		0.758		
Male				
Female				
**Race**		0.189		
White				
Others				
**Laterality**		0.051		0.115
Unilateral				
Bilateral				
Unknown				
**Primary site**		0.004		0.15
Retina	Ref			
Choroid	0.156(0.047–0.52)			
Ciliary body	0.286(0.124–0.662)			
Vitreous	0.288(0.137–0.606)			
**Pathological type**		< 0.001		0.008
DLBCL	Ref		Ref	
MALT	0.144(0.065–0.322)		0.233(0.101–0.534)	
NHL, NOS	0.587(0.325–1.062)		0.823(0.439–1.543)	
Other/unclassified^b^	0.59(0.361–0.964)		0.813(0.489–1.353)	
**Ann arbor stage**		0.057		
I to II				
III to IV				
Unknown				
**Surgery**		0.441		
NO				
Performed				
**Radiotherapy**		0.362		
No/unknown				
Performed				
**Chemotherapy**		< 0.001		0.003
No/unknown	Ref		Ref	
Performed	0.415(0.273–0.632)		0.498(0.316–0.785)	

## Discussion

Considering the rarity of PIOL, few population-based studies of PIOL have been conducted. The current study used the SEER database, a prominent resource for research on rare malignancies, to conduct this population-based cohort study of PIOL and obtain an in-depth understanding of this disease.

This study found that the overall age-adjusted incidence of PIOL in the US was 0.23, with an upward trend over three decades, and the incidence in 2018 was 0.27. The increasing trend may be attributed to the increased numbers of immunodeficient and immunosuppressed patients, prolonged life expectancy, and advances in diagnostic methods [15].

In line with previous studies [26], this study revealed a mean age at diagnosis of 62.5 years. The youngest patient in this study was only 8 years old; such a young age is extremely rare in PIOL. The incidence of PIOL in individuals aged ≥ 60 years was nearly 18 times higher than in those aged < 60 years. Meanwhile, advanced age was related to worse DSS. Elderly people often have more comorbidities than younger individuals do and so cannot endure intensive treatments with high toxicity; this has an adverse effect on prognosis [27, 28]. Consistent with previous studies, DLBCL was the most common histological subtype of PIOL, followed by MALT lymphoma, and DLBCL was more common in the elderly than in younger individuals, which may affect the survival time of these patients [5, 11, 29, 30].

Compared with lymphomas in other primary sites of PIOL, those in the choroid and ciliary body have better prognoses, which is mainly due to the radiation sensitivity and less aggressive clinical course of those sites [31–33]. Meanwhile, lymphomas in the retina and vitreous were associated with poor prognosis because they usually present as high-grade lymphomas and are often associated with CNS lymphoma [33, 34]. Unilateral PIOL did not correlate with better survival and may require positive treatments, similar to patients with bilateral disease, which conforms with the guidelines of the British Neuro-Oncology Society [35].

There are no uniform treatment protocols or guidelines for PIOL. Available treatments aim to remit the intraocular disease to preserve patients’ visual acuity and prevent CNS involvement, which is a major cause of death in PIOL patients [5, 18]. The comprehensive treatment strategies for PIOL include local treatment, such as ocular radiotherapy and intravitreal chemotherapy; systemic treatment, mainly depending on high doses of methotrexate; and a combination of both. The International Primary Central Nervous System Lymphoma Collaborative Group (IPCG) recommends local therapy for unilateral PIOL. If both eyes are involved, there is still a preference for local treatment, and systemic treatment should also be applied, if necessary [5]. However, the British Neuro-Oncology Society suggests ocular irradiation combined with systemic chemotherapy [36].

Radiation was the most commonly used therapy in our study. The radiation regimen for lymphoma localized in the eye usually varies from 30 to 45 Gy in approximately 15 fractions, and radiation should be performed in both eyes because PIOL always develops bilaterally [5, 37]. Although ocular irradiation may cause cataract, radiation retinopathy, or optic neuropathy, its benefits outweigh its complications [8, 36].

Intravitreal chemotherapy was proposed and used as a local treatment for PIOL in the 1990s, which improved the treatment outcomes of PIOL and decreased its morbidity [38]. Methotrexate (dose, 400 μg in 0.1 ml) is the main drug for intravitreal chemotherapy, and the number of total injections can be modulated according to the patient’s clinical response [39–41]. A single-center retrospective study in China including 16 patients (28 eyes) with intraocular lymphoma reported that local therapy may preserve visual acuity [42]. Rituximab has also been used for intravitreal injections, which may necessitate fewer injections and entail lower toxicity [43–45].

Systemic chemotherapy with methotrexate, a treatment mainly based on experience with PCNSL, is considered when the disease develops in both eyes or involves the CNS [18, 46]. However, the efficacy of combination therapy, namely, systemic chemotherapy combined with local treatment, to decrease the risk of CNS relapse remains controversial due to the inconsistent results of retrospective studies on PIOL [17, 18]. The IPCG analyzed the treatment outcomes in PCNSL patients with ocular involvement and concluded that ocular treatment prolonged disease control, but did not affect the prognosis or ocular recurrence risk [47]. Other studies also reported that local treatment was effective in eliminating tumor cells in the eyes, but it could not control CNS relapse [18]. However, a meta-analysis of 83 studies suggested that intravitreal injection combined with systemic chemotherapy could prolong survival in patients with CNS involvement, and that combining it with radiotherapy further reduced recurrence and mortality rates [48].

Via multivariable Cox regression analysis, our study identified chemotherapy as an independent risk factor for prognosis. Our additional analyses revealed that the proportion of DLBCL in patients who received chemotherapy was high (*P* < 0.001), which might have caused biases [18, 48]. Different chemotherapeutic modalities may affect the prognosis of PIOL patients; however, detailed information on chemotherapy cannot be extracted from the SEER database. Thus, further analysis of chemotherapy is not feasible.

The survival of PIOL patients has been improving, partly due to the development of diagnostic tools, including imaging, blood testing, immunocytological/histological evaluation, biochemical analysis, and more optimal diagnostic panels, namely, the combination of cytologic smears, immunohistochemistry, and cytokine analysis [49–51]. Melphalan, temozolomide, lenalidomide with or without rituximab, and ibrutinib have also shown promising results for PIOL [52–56].

This study had some limitations. First, this retrospective study was based on the SEER data, which might have caused unavoidable biases. Due to the rarity of PIOL, a large prospective study seems impractical. Second, detailed data on radiotherapy administration protocols, chemotherapy regimens, and surgical approaches are missing from the SEER database. Thus, specific treatment regimens could not be accurately determined. Third, the SEER program provided limited information on the extension of lymphoma in PIOL patients, and CNS progression is believed to prominently affect the survival of these patients. Fourth, the SEER database does not have a specialized code for the vitreous, so this study used the site “eye, NOS” to denote the vitreous, because other sites have separate codes and this particular site has been considered the vitreous in previous studies [57]. Overall, these limitations are common in studies based on SEER data. Nonetheless, the SEER remains a significant source for studying rare tumors, taking these limitations into account. The present study provides important insights for PIOL and valuable information on the incidence, prognostic factors, and survival outcomes in PIOL.

## Conclusions

The present study shows that PIOL is a rare type of lymphoma with a poor prognosis and an increasing incidence trend. It mostly affects individuals aged ≥ 60 years, and DLBCL is its most common pathological type. For PIOL patients, survival analysis showed that age < 60 years and non-DLBCL pathological types are associated with good survival. The survival of patients with PIOL has improved over years.

## Data Availability

Data can be extracted from the SEER database after completing their agreement form and requesting access from their website (https://seer.cancer.gov).

## Abbreviations

PIOL: Primary intraocular lymphoma
PCNSL: Primary central nervous system lymphoma
CNS: Central nervous system
DLBCL: Diffuse large B-cell lymphoma
IL: Interleukin
SEER: Surveillance, Epidemiology, and End Results
AAS: Ann Arbor stage
APC: Annual percentage change
CI: Confidence interval
DSS: Disease-specific survival
MALT: Mucosa-associated lymphoid tissue
NHL: Non-Hodgkin lymphoma
IPCG: International Primary Central Nervous System Lymphoma Collaborative Group
