# Correction: Epidemiology and survival outcomes of patients with primary intraocular lymphoma: a population-based analysis

**DOI:** 10.1186/s12886-023-02781-z

**Published:** 2023-02-09

**Authors:** Lin-feng He, Jin-di Zhang, Xin-xin Chen, Rui-li Wei

**Affiliations:** Department of Ophthalmology, Changzheng Hospital of Naval Medicine University, 415 Fengyang Road, Shanghai, China

**Correction: BMC Ophthalmol 22, 486 (2022)**

**https://doi.org/10.1186/s12886-022-02702-6**

Following publication of the original article [1], the author group identified errors in Fig. 5b and c and in Tables 2, 3 and 4. Two errors were also noted in the Results and Discussion sections.

The correct version of the figures and tables as well as the updated Results and Discussion sections are given below.

Fig. 5b 
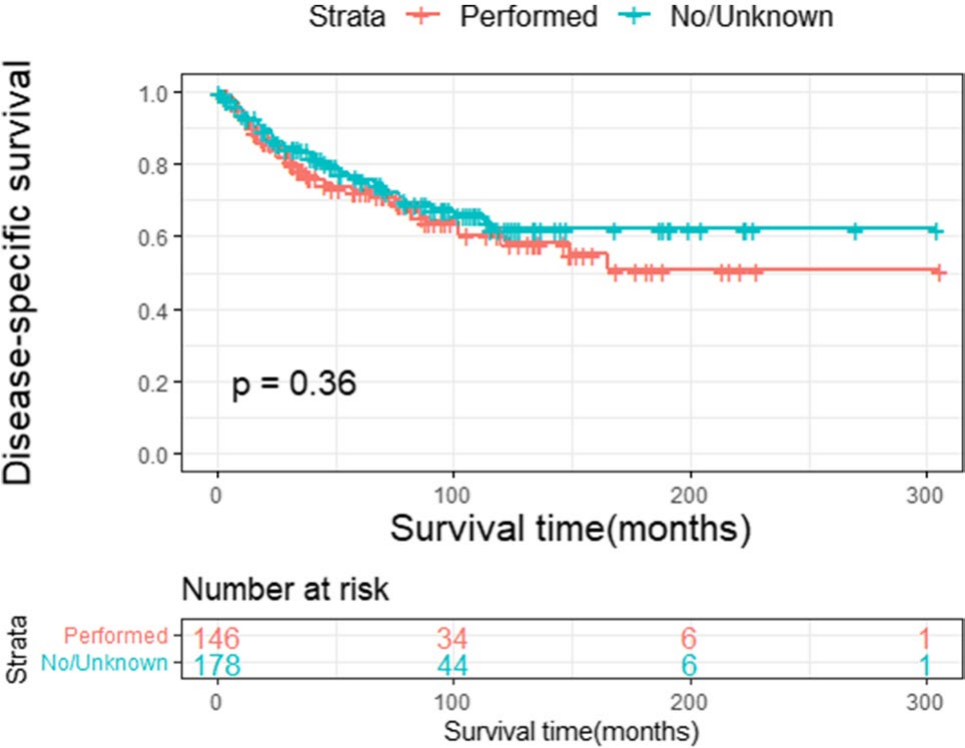


Fig. 5c 
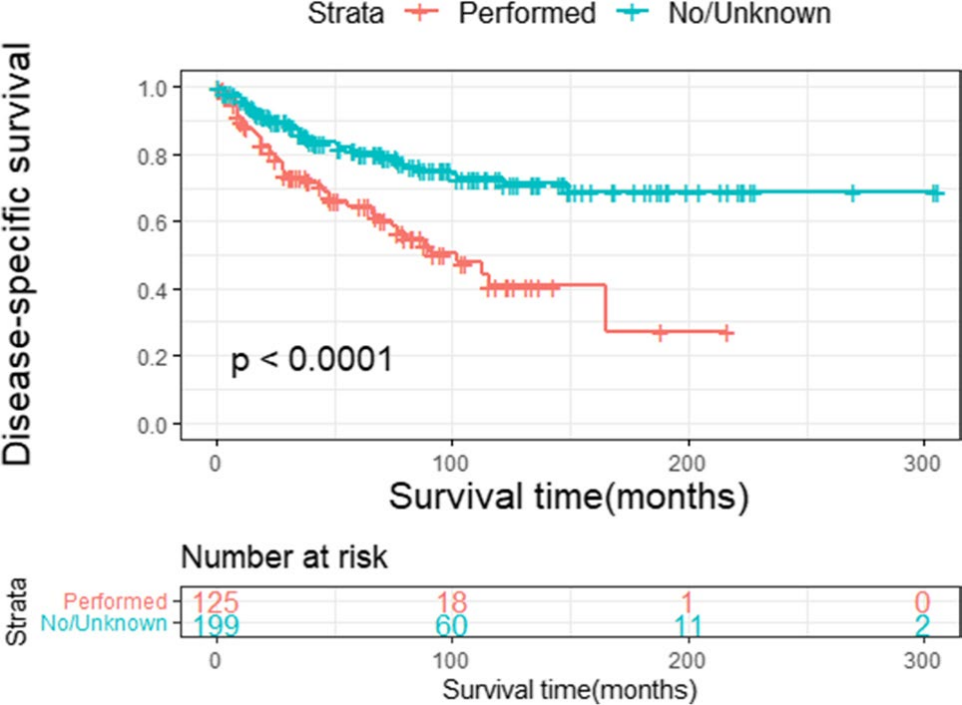


**Table 2:** Demographic and clinical characteristics of patients with primary intraocular lymphoma

Variables	Total	<60	≥60	*P*
Number of patients(%)	326	91 (27.9)	235 (72.1)	
**Age**				
Mean (SD)	66.1 (14.2)	48.5 (10.4)	72.9 (8.6)	
Median [Min, Max]	67.5 [8,97]	52 [8,59]	72 [60-97]	
**Year of diagnosis**				0.059
1992-2002	76 (23.3)	18 (19.8)	58 (24.7)	
2003-2012	138 (42.3)	48 (52.7)	90 (38.3)	
2013-2018	112 (34.4)	25 (27.5)	87 (37)	
**Sex**				0.208
Male	129 (39.6)	41 (45.1)	88 (37.4)	
Female	197 (60.4)	50 (54.9)	147 (62.6)	
**Race**				0.105
White	275 (84.4)	72 (79.1)	203 (86.4)	
Others^a^	51 (15.6)	19 (20.9)	32 (13.6)	
**Laterality**				0.202
Unilateral	274 (84)	78 (85.7)	196 (83.4)	
Bilateral	44 (13.5)	13 (14.3)	31 (13.2)	
Unknown	8 (2.5)		8 (3.4)	
**Primary site**				0.185
Retina	12 (3.7)		12 (5.1)	
Choroid	24 (7.4)	7 (7.7)	17 (7.2)	
Ciliary body	66 (20.2)	19 (20.9)	47 (20)	
Vitreous	224 (68.7)	65 (71.4)	159 (67.7)	
**Pathological type**				0.003
DLBCL	99 (30.4)	18 (19.8)	81 (34.5)	
MALT	88 (27)	37 (40.7)	51 (21.7)	
NHL, NOS	56 (17.2)	13 (14.3)	43 (18.3)	
Other/unclassified	83 (25.5)	23 (25.3)	60 (25.5)	
**Ann arbor stage**				0.839
I to II	209 (64.1)	58 (63.7)	151 (64.3)	
III to IV	31 (9.5)	10 (11)	21 (8.9)	
Unknown	86 (26.4)	23 (25.3)	63 (26.8)	
**Surgery**				0.153
No/unknown	232 (71.2)	70 (76.9)	162 (68.9)	
Performed	94 (28.8)	21 (23.1)	73 (31.1)	
**Radiotherapy**				0.757
No/unknown	180 (55.2)	49 (53.8)	131 (55.7)	
Performed	146 (44.8)	42 (46.2)	104 (44.3)	
**Chemotherapy**				0.978
No/unknown	201 (61.7)	56 (61.5)	145 (61.7)	
Performed	125 (38.3)	35 (38.5)	90 (38.3)	

Others^a^: Black, American Indian/AK Native, Asian/Pacific Islander, and unknown

Other/unclassified^b^: Malignant lymphoma, Mantle cell lymphoma, Burkitt lymphoma, Follicular lymphoma, Peripheral T-cell lymphoma, Anaplastic large cell lymphoma, Chronic lymphocytic leukemia/small lymphocytic lymphoma

*NHL* non–Hodgkin lymphoma, *NOS* not otherwise specified

**Table 3:** The results of the log-rank test

Variables	Total	<60	≥60
**Age**	<0.001		
<60			
≥60			
**Year of diagnosis**	0.006	0.329	0.035
1992-2002			
2003-2012			
2013-2018			
**Sex**	0.757	0.4	0.794
Male			
Female			
**Race**	0.188	0.091	0.785
White			
Others			
**Laterality**	0.05	0.03	0.072
Unilateral			
Bilateral			
Unknown			
**Primary site**	0.002	0.946	0.018
Retina			
Choroid			
Ciliary body			
Vitreous			
**Pathological type**	<0.001	0.347	<0.001
DLBCL			
MALT			
NHL, NOS			
Other/unclassified^b^			
**Ann arbor stage**	0.057	0.22	0.032
I to II			
III to IV			
Unknown			
**Surgery**	0.44	0.949	0.521
No/unknown			
Performed			
**Radiotherapy**	0.361	0.147	0.715
No/unknown			
Performed			
**Chemotherapy**	<0.001	0.001	0.001
No/unknown			
Performed			

**Table 4:** The results of the univariate and multivariate Cox regression analysis

Variables	Univariate analysis		Multivariate analysis	
	HR (95% CI)	*P* value	HR (95% CI)	*P* value
**Age**		<0.001		<0.001
<60	Ref		Ref	
≥60	3.508 (1.904-6.464)		3.146 (1.699-5.826)	
**Year of diagnosis**		0.008		0.054
1992-2002	Ref			
2003-2012	0.524 (0.333-0.826)			
2013-2018	0.473 (0.251-0.89)			
**Sex**		0.758		
Male				
Female				
**Race**		0.189		
White				
Others				
**Laterality**		0.051		0.115
Unilateral				
Bilateral				
Unknown				
**Primary site**		0.004		0.15
Retina	Ref			
Choroid	0.156 (0.047-0.52)			
Ciliary body	0.286 (0.124-0.662)			
Vitreous	0.288 (0.137-0.606)			
**Pathological type**		<0.001		0.008
DLBCL	Ref		Ref	
MALT	0.144 (0.065-0.322)		0.233 (0.101-0.534)	
NHL, NOS	0.587 (0.325-1.062)		0.823 (0.439-1.543)	
Other/unclassified^b^	0.59 (0.361-0.964)		0.813 (0.489-1.353)	
**Ann arbor stage**		0.057		
I to II				
III to IV				
Unknown				
**Surgery**		0.441		
Performed				
No/unknown				
**Radiotherapy**		0.362		
Performed				
No/unknown				
**Chemotherapy**		<0.001		0.003
Performed	Ref		Ref	
No/unknown	0.415 (0.273-0.632)		0.498 (0.316-0.785)	

Error in Results section

On page 5, the second paragraph of the first column reads:

“The whole cohort was analyzed using log-rank tests and univariate Cox proportional hazards models, which revealed that age, years of diagnosis, laterality, primary site, pathological type, and chemotherapy had an effect on DSS.”

The sentence should read:

“The whole cohort was analyzed using log-rank tests and univariate Cox proportional hazards models, which revealed that age, years of diagnosis, primary site, pathological type, and chemotherapy had an effect on DSS.”

Error in Discussion section

On page 6, the first sentence of the first paragraph currently reads:

“In line with previous studies [2] (reference 26 in the original article], this study revealed a mean age at diagnosis of 62.5 years.”

The sentence should read:

“In line with previous studies [2] (reference 26 in the original article], this study revealed a mean age at diagnosis of 66.1 years.”

The original article [1] has been corrected.

The authors apologise for these errors.
